# Transcriptome and translatome profiles of *Streptomyces* species in different growth phases

**DOI:** 10.1038/s41597-020-0476-9

**Published:** 2020-05-08

**Authors:** Woori Kim, Soonkyu Hwang, Namil Lee, Yongjae Lee, Suhyung Cho, Bernhard Palsson, Byung-Kwan Cho

**Affiliations:** 1 Department of Biological Sciences and KI for the BioCentury, Korea Advanced Institute of Science and Technology, Daejeon, 34141 Republic of Korea; 2 Department of Bioengineering, University of California San Diego, La Jolla, CA 92093 USA; 3 Department of Pediatrics, University of California San Diego, La Jolla, CA 92093 USA; 4 Novo Nordisk Foundation Center for Biosustainability, Technical University of Denmark, Lyngby, 2800 Denmark; 5 Intelligent Synthetic Biology Center, Daejeon, 34141 Republic of Korea

**Keywords:** Prokaryote, Next-generation sequencing, RNA sequencing

## Abstract

*Streptomyces* are efficient producers of various bioactive compounds, which are mostly synthesized by their secondary metabolite biosynthetic gene clusters (smBGCs). The smBGCs are tightly controlled by complex regulatory systems at transcriptional and translational levels to effectively utilize precursors that are supplied by primary metabolism. Thus, dynamic changes in gene expression in response to cellular status at both the transcriptional and translational levels should be elucidated to directly reflect protein levels, rapid downstream responses, and cellular energy costs. In this study, RNA-Seq and ribosome profiling were performed for five industrially important *Streptomyces* species at different growth phases, for the deep sequencing of total mRNA, and only those mRNA fragments that are protected by translating ribosomes, respectively. Herein, 12.0 to 763.8 million raw reads were sufficiently obtained with high quality of more than 80% for the Phred score Q30 and high reproducibility. These data provide a comprehensive understanding of the transcriptional and translational landscape across the *Streptomyces* species and contribute to facilitating the rational engineering of secondary metabolite production.

## Abstract (summary)

**Table:** 

Measurement(s)	transcriptome • translation • translatome
Technology Type(s)	RNA sequencing • Ribo-Seq
Factor Type(s)	Growth phases
Sample Characteristic - Organism	Streptomyces avermitilis • Streptomyces clavuligerus • Streptomyces lividans • Streptomyces venezuelae • Streptomyces tsukubensis

Machine-accessible metadata file describing the reported data: 10.6084/m9.figshare.12045603

## Background & Summary

*Streptomyces*, which comprise the largest genus of Actinobacteria, are huge natural reservoir of secondary metabolites, including antibiotics, immunosuppressants, and other medicinal compounds^1–6^. Recent advancements in high-throughput sequencing have led to the development of the genome mining approach, which implicates that the genome of each *Streptomyces* species has more than 30 secondary metabolite biosynthetic gene clusters (smBGCs) with potential to produce various unexplored secondary metabolites^2^. These secondary metabolites are synthesized by a series of enzymatic reactions, which depend on the supply of precursor molecules from primary metabolism, such as acetyl-coenzyme A and amino acids^7^. After active growth terminates, an overall metabolic transition occurs, which leads to the activation of secondary metabolite production^8,9^; this metabolic transition from primary to secondary metabolism is governed by multi-layered regulatory mechanisms at transcriptional, translational, and post-translational levels^10,11^. Thus, understanding the complex regulatory systems of the metabolic transition is important to enhance secondary metabolite production. The overall metabolic transition encompasses diverse genome-wide gene expression changes, which are regulated by signaling cascades from the pleiotropic regulators to pathway-specific regulators^8,10,12,13^. To understand the underlying molecular mechanisms of metabolic transitions, transcriptional changes that occur between growth phases have been studied^13–15^. For example, the time-series transcriptome analysis of *Streptomyces coelicolor* demonstrated that coherent genes that are involved in specific metabolism and their regulatory genes exhibit similar expression patterns during metabolic transitions; this suggests that primary metabolism-related genes are functionally connected to the smBGC genes through regulatory gene expression. Based on this suggestion, putative regulatory genes and their interconnected networks could be identified by screening genes that have similar expression patterns^13^.

Bacteria can fine-tune gene expression both at the transcriptional and translational levels^16,17^. For example, *Escherichia coli* proteome analysis revealed that only approximately half of protein abundance is determined by transcriptional regulation, which indicates the existence of various post-transcriptional regulation^18^. In this regard, deciphering translational dynamics is important to understanding post-transcriptional regulations that are closely related to cellular protein levels^19^. Recently, ribosome profiling has been used to measure translational levels by deep sequencing of the ribosome-protected mRNA fragments (RPFs) at the position of the translating ribosome^20^. Several ribosome profiling studies in *Streptomyces* have been reported by our research group for *S. coelicolor*, *S. clavuligerus*, and *S. lividans*, which revealed translational buffering of secondary metabolism-related genes at a later growth phase and that translational abundance is more consistently maintained than transcript abundance^11,21,22^. Translational regulations are advantageous for the tight control of secondary metabolite biosynthesis, as translation requires the highest energy costs among all cellular reactions^19^. Moreover, the expression of smBGC-associated genes can be more rapidly regulated at the translational level than at the transcriptional level in response to dynamic environmental changes^23^. Given the dynamic relationship between transcription and translation, as exhibited by translational buffering^11^, integrative analysis at both levels should unravel complex regulations in *Streptomyces*. However, transcriptomic and translatomic data have covered only a small portion of approximately 350 reported *Streptomyces* genomes, which have not been systematically validated at the multi-species level.

**Fig. 1:**
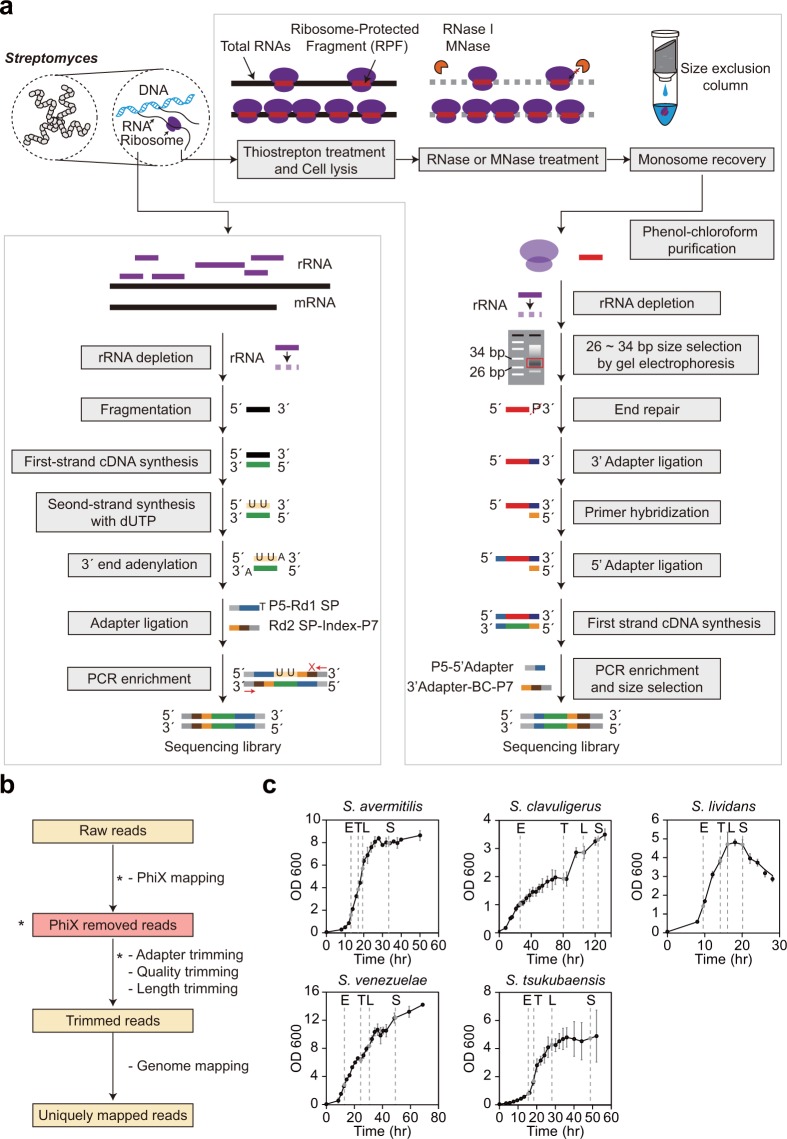
Overall flow of RNA-Seq and ribosome profiling data construction of five *Streptomyces* species. (**a**) The sequencing library construction protocol for RNA-Seq and ribosome profiling. P5 and P7 were the PCR primers, Rd1 SP and Rd2 SP were the sequencing primers, and BC was the barcode sequence. (**b**) An overview of processing and mapping of the sequencing reads. The criteria or parameters are shown. The steps indicated with asterisk (*) are performed only for the ribosome profiling data. (**c**) The growth profile of five *Streptomyces* species in R5− medium. Sampling time points are represented by a grey dot, which are the early-exponential (E), transition (T), late-exponential (L), and stationary (S) points.

In this study, we provide RNA-Seq and ribosome profiling data of five *Streptomyces* species at four different growth phases, followed by validation of the read quality. The species were *S. avermitilis* MA-4680, *S. clavuligerus* ATCC27064, *S. lividans* TK24, *S. venezuelae* ATCC15439, and *S. tsukubaensis* NRRL 18488, which are industrial strains that produce antifungal avermectin, β-lactamase inhibitor clavulanic acid, and immunosuppressant FK506, respectively^24–26^. *S. lividans* and *S. venezuelae* were characterized by their fast growth and ease of genetic manipulation, and have been employed as heterologous expression hosts^27–30^. An overview of the preparation of transcriptomic and translatomic data is illustrated in Fig. 1. A total of 12 to 83.5 million raw reads for RNA-Seq and 113 to 763.8 million raw reads for ribosome profiling were obtained. Although the RNA-Seq and ribosome profiling data of two species (*S. clavuligerus*^21^ and *S. lividans*^22^) among the five species were already reported in previous studies by our research group, this study provided a uniformly processed and mapped dataset of all five species. This facilitates the efficiency of the comparative transcriptome and translatome analysis at multi-time points between multi-species. Further, understanding the transcriptional and translational regulatory mechanisms and developing regulatory synthetic parts, such as promoters, ribosome-binding sequences, 5′ untranslated regions, and terminators^4^ from the dataset allows rational genome engineering for efficient secondary metabolite production by *Streptomyces*^11^.

## Methods

### Strains and cell growth

*Streptomyces* strains were inoculated from their 20% glycerol stock of spores into 50 mL of R5− liquid medium with 8 g of glass beads (3 ± 0.3 mm diameter) in a 250 mL baffled flask, grown at 30 °C, and pre-cultured at 250 rpm. The R5− liquid medium consists of 103 g L^−1^ sucrose, 0.25 g L^−1^ K_2_SO_4_, 10.12 g L^−1^ MgCl_2_∙6H_2_O, 10 g L^−1^ glucose, 0.1 g L^−1^ casamino acids, 5 g L^−1^ yeast extract, 5.73 g L^−1^ TES (pH 7.2), 0.08 mg L^−1^ ZnCl_2_, 0.4 mg L^−1^ FeCl_3_∙6H_2_O, 0.02 mg L^−1^ CuCl_2_∙2H_2_O, 0.02 mg L^−1^ MnCl_2_∙4H_2_O, 0.02 mg L^−1^ Na_2_B_4_O_7_∙10H_2_O, 0.02 mg L^−1^ (NH_4_)_6_Mo_7_O_24_∙4H_2_O, and 0.28 g L^−1^ NaOH. The grown mycelium was inoculated to fresh R5− medium with an initial optical density of 0.05 at 600 nm for the main culture as biological duplicates and grown under the previously mentioned conditions. The cells were sampled at four different time points based on the growth profile of each strain, as follows: early-exponential (E), transition (T), late-exponential (L), and stationary (S) phases. The E, T, L, and S time points were 13, 17, 19.5, and 33.5 h for *S. avermitilis*, 26, 80, 105.5, and 125 h for *S. clavuligerus*, 9.5, 14, 16, and 20 h for *S. lividans*, 12.5, 24.5, 30.5, and 48.5 h for *S. venezuelae*, and 15, 18.5, 28, and 48 h for *S. tsukubaensis* after inoculation, respectively (Fig. 1c). At the sampling time points for the ribosome profiling samples, thiostrepton (Sigma-Aldrich, St. Louis, MO, USA) was added to the cultures to a final concentration of 20 μM to compartment the translating ribosomes on the mRNA, which is a highly sensitive drug for *Streptomyces* compared to chloramphenicol or other drugs^31,32^. The cultures were then incubated for 5 min at 30 °C, and subsequently harvested for the construction of ribosome profiling libraries.

### RNA-Seq library preparation and high-throughput sequencing

The overview of the library construction of RNA-Seq is illustrated in Fig. 1a. The harvested cells were washed with polysome buffer (20 mM Tris-HCl, pH 7.5; 140 mM NaCl and 5 mM MgCl_2_), and then resuspended with 500 μL lysis buffer (0.3 M sodium acetate, pH 5.2; 10 mM ethylenediaminetetraacetic acid and 1% Triton X-100). The resuspended cells were frozen with liquid nitrogen and grounded using a mortar and pestle. The ground mycelium was thawed and centrifuged at 4 °C for 10 min at 16,000 × *g*. The supernatant was collected and stored at −80 °C. Following the preparation of lysates from four growth phases as biological duplicates, the lysates were mixed with a solution of phenol:chloroform:isoamyl alcohol (25:24:1, v/v), and the mixtures were separated by centrifugation. DNA in the extracted RNA samples were removed by treatment with 2 μL DNase I (NEB, Ipswich, MA, USA), 5 μL 10 × DNase I buffer, and 1 μL SUPERase-In RNase Inhibitor (Thermo Scientific, Waltham, MA, USA). Lastly, the DNase I-treated RNA samples were purified using phenol:chloroform:isoamyl alcohol (25:24:1, v/v) and ethanol precipitation. To eliminate rRNAs in the recovered RNA samples, the Ribo-Zero rRNA Removal Kit for Bacteria (Epicentre, Madison, WI, USA) was used according to the manufacturer’s instructions. The quality of rRNA-depleted RNA samples was checked using 2% agarose gel electrophoresis. The suitable RNA samples were then used to construct RNA sequencing libraries using the TruSeq Stranded mRNA Library Prep Kit (Illumina, San Diego, CA, USA). The size distributions of the final libraries were checked using the Agilent 2200 TapeStation System (Agilent, Santa Clara, CA, USA). The constructed libraries were sequenced on the HiSeq. 2500 platform using either a 100-bp (*S. lividans*, *S. avermitilis*, *S. clavuligerus*, and *S. venezuelae*) or 50-bp (*S. tsukubaensis*) single-end read recipe (Fig. 1a).

### Data processing of RNA-Seq reads

Raw FASTQ files were processed using the CLC Genomics Workbench (CLC Bio, Aarhus, Denmark). Raw reads were trimmed by their overall quality (score: 0.05; maximum ambiguous nucleotides: (2) and length (minimum length: 15 nucleotides). The filtered reads were mapped to each reference genome sequence with the default parameters (mismatch cost: 2; insertion cost: 2; deletion cost: 3; length fraction: 0.9; similarity fraction: 0.9; and ignore non-specific matches). The accession number of each reference genome is as follows: *S. avermitilis* MA-4680 (NC_010572), *S. clavuligerus* ATCC27064 (chromosome NZ_CP027858, plasmid NZ_CP027859), *S. lividans* TK24 (NZ_CP009124), *S. venezuelae* ATCC15439 (CP013129), and *S. tsukubaensis* NRRL18488 (chromosome CP020700, plasmid CP020701, and CP020702). The statistics pertaining to quality trimming and reference mapping are summarized in Table 1. The number of uniquely mapped reads to each gene were counted using the RNA-Seq analysis tool in the CLC Genomics Workbench and the read counts were normalized using the DESeq. 2 package in R^33^.

**Table 1:** Overall statistics of RNA-Seq data.

Species	Growth phase	Number of raw reads	Average length (bp)	Number of trimmed_read	Percentage trimmed	Trimmed reads length (bp)	Number of randomly mapped reads	Number of uniquely mapped reads	Percentage of uniquely mapped reads (%)	Raw read FASTQ accession
*S. avermitilis* MA-4680	E1	15,222,700	101	15,222,324	100.00	100.9	14,743,001	14,475,094	95.09	SRP158023
	E2	15,540,304	101	15,539,540	100.00	100.8	14,842,965	14,232,752	91.59	
	T1	18,962,695	101	18,961,958	100.00	100.8	17,584,931	16,820,053	88.70	
	T2	18,054,983	101	18,054,302	100.00	100.9	16,337,750	14,948,455	82.80	
	L1	13,904,005	101	13,903,462	100.00	100.9	12,858,182	12,238,050	88.02	
	L2	16,814,305	101	16,813,651	100.00	100.8	15,778,544	15,212,127	90.47	
	S1	16,662,552	101	16,661,924	100.00	100.9	15,627,234	14,761,494	88.59	
	S2	16,278,766	101	16,278,123	100.00	100.9	14,643,706	12,519,514	76.91	
*S. clavuligerus* ATCC 27064	E1	14,798,628	101	14,798,315	100.00	100.8	11,098,664	9,036,995	61.07	SRP188290
	E2	14,979,238	101	14,978,853	100.00	100.8	10,822,676	8,622,479	57.56	
	T1	15,701,669	101	15,701,289	100.00	100.8	10,501,955	9,056,077	57.68	
	T2	12,420,952	101	12,420,654	100.00	100.8	10,776,124	10,096,097	81.28	
	L1	13,207,846	101	13,207,520	100.00	100.8	7,770,986	7,283,393	55.15	
	L2	13,782,302	101	13,782,042	100.00	100.9	7,706,785	7,193,337	52.19	
	S1	13,526,270	101	13,525,948	100.00	100.8	12,683,457	12,292,000	90.88	
	S2	13,272,332	101	13,272,058	100.00	100.6	12,663,763	11,921,210	89.82	
*S. lividans* TK24	E1	15,062,705	101	15,062,394	100.00	100.9	13,098,717	12,182,999	80.88	PRJEB31507
	E2	15,941,901	101	15,941,640	100.00	100.9	14,010,897	12,726,791	79.83	
	T1	14,403,255	101	14,402,994	100.00	100.9	12,594,960	11,858,708	82.34	
	T2	15,701,759	101	15,701,526	100.00	100.9	14,333,364	13,320,933	84.84	
	L1	16,081,294	101	16,080,979	100.00	100.8	14,679,573	14,003,911	87.08	
	L2	15,402,577	101	15,402,313	100.00	100.8	13,896,464	12,784,443	83.00	
	S1	15,650,348	101	15,650,033	100.00	100.9	14,016,141	12,866,712	82.22	
	S2	17,244,360	101	17,243,710	100.00	100.9	13,310,075	10,371,378	60.15	
*S. venezuelae* ATCC15439	E1	13,343,482	101	13,339,752	99.97	100.9	11,002,160	9,468,993	70.98	PRJEB34219
	E2	13,150,521	101	13,147,020	99.97	100.9	10,562,003	9,986,725	75.96	
	T1	14,479,417	101	14,474,269	99.96	100.9	13,219,134	12,480,953	86.23	
	T2	12,310,427	101	12,307,770	99.98	100.9	10,406,022	9,456,882	76.84	
	L1	12,192,708	101	12,173,069	99.84	100.9	10,371,418	9,415,019	77.34	
	L2	12,728,235	101	12,723,109	99.96	100.9	10,435,448	8,569,124	67.35	
	S1	13,022,122	101	13,019,964	99.98	100.9	10,770,484	9,268,463	71.19	
	S2	11,969,031	101	11,957,872	99.91	100.9	9,138,846	8,090,654	67.66	
*S. tsukubaensis NRRL18488*	E1	41,652,947	51	41,627,595	99.94	50.9	41,292,669	31,773,092	76.33	SRP103795
	E2	35,401,018	51	35,382,993	99.95	50.8	34,839,050	34,058,311	96.26	
	T1	53,758,514	51	53,721,965	99.93	50.8	52,441,945	51,123,244	95.16	
	T2	25,432,836	51	25,421,462	99.96	50.8	24,909,553	24,095,211	94.78	
	L1	83,469,019	51	83,456,281	99.98	50.6	82,904,135	76,980,981	92.24	
	L2	39,371,004	51	39,339,183	99.92	50.8	38,739,771	36,079,744	91.71	
	S1	78,596,694	51	78,587,491	99.99	50.6	77,714,238	61,851,605	78.70	
	S2	51,475,167	51	51,441,666	99.93	50.9	50,553,226	44,055,753	85.64	

### Ribosome profiling library preparation and high-throughput sequencing

An overview on the library construction of ribosome profiling is illustrated in Fig. 1a ^21^. The mycelium that was treated by thiostrepton was collected by centrifugation at 4 °C for 10 min at 3,000 × *g*, and the cell pellet was washed with 2 mL of polysome buffer that was composed of 20 mM Tris-HCl (pH 7.5), 140 mM NaCl, and 5 mM MgCl_2_ with 20 μM thiostrepton. The washed pellet was re-suspended in 1 mL of lysis buffer composed of 950 μL of polysome buffer and 50 μL of 20% Triton X-100 with 20 μM thiostrepton. The resuspended cells were dripped into a mortar filled with liquid nitrogen and then grounded with a pestle. The cell debris was removed by centrifugation at 4 °C for 5 min at 3,000 × *g*. The supernatant was further clarified and collected by centrifugation at 4 °C for 10 min at 16,000 × *g*. To digest RNA in the lysate (containing 50 μg RNA), the *S. avermitilis* and *S. tsukubaensis* samples were treated with 750 U of RNase I (Invitrogen, Waltharn, MA, USA) at 37 °C for 45 min, and the remaining strains were treated with 400 U of Micrococcal Nuclease (MNase) (NEB), 20 μl of 10× MNase buffer, and 2 μl of 100× Bovine Serum Albumin (BSA) (NEB) at 37 °C for 2 h. The samples were then loaded onto Illustra MicroSpin S-400 HR Columns (GE Healthcare Life Sciences, Marlborough, MA, USA) that were previously washed three times with 500 μL of washing buffer, which was composed of 50 mM Tris-HCl (pH 8.0), 250 mM NaCl, 50 mM MgCl_2_, 25 mM EGTA, and 1% Triton X-100. The column was centrifuged at 4 °C for 2 min at 400 × *g*, and the flow-through was further purified by a phenol-chloroform-isoamyl alcohol extraction and ethanol precipitation. rRNA was depleted with the Ribo-Zero rRNA Removal Kit (Epicentre) according to the manufacturer’s instructions. The ribosome-protected RNA fragments (RPF) of between 26 and 34 bp were separated by electrophoresis for 65 min at 200 V using 15% polyacrylamide TBE-urea gel (Invitrogen), and eluted in 400 μL of RNA gel extraction buffer, which was composed of 300 mM sodium acetate pH 5.5, 1 mM EDTA, and 0.25% (w/v) SDS. The samples were frozen for 30 min at −80 °C and then incubated at 37 °C for 4 h. The eluted RNAs were isolated by ethanol precipitation and purified once again with the RNeasy MinElute Column (Qiagen, Hilden, Germany) using the manufacturer’s protocol. The enriched RPFs were then denatured for 90 s at 80 °C and incubated for 1 h at 37 °C with 5 mL of 10× T4 Polynucleotide Kinase (PNK) buffer (NEB), 20 U of SUPERase-In RNase Inhibitor, and 10 U of T4 PNK (NEB) to dephosphorylate the 3′ end. The dephosphorylated RNAs were purified using the RNeasy MinElute Column (Qiagen). The sequencing library was constructed from the end-repaired RPFs using the NEBNext Multiplex Small RNA Library Prep Set for Illumina (NEB) according to the manufacturer’s instructions. The final library of approximately 150–160 bp was size-selected by gel electrophoresis for 90 min at 100 V using a 2% agarose gel that was dyed with SYBR Gold Nucleic Acid Gel Stain (Bio-Rad, Hercules, CA, USA). The concentration of the final library was measured using a Qubit 2.0 Fluorometer (Invitrogen) and the Qubit dsDNA HS Kit. The size distribution was assessed using the Agilent 2200 TapeStation System (Agilent). The constructed library was sequenced on the Illumina HiSeq. 2500 platform using the 50-bp single-end read recipe (Fig. 1b).

### Data processing of ribosome profiling reads

The libraries of seven samples of *S. avermitilis—*except for the E1 sample—and six samples of *S. venezuelae—*except for the T2 and S2 samples—were prepared and sequenced twice to increase the output and merged for further data processing (Table 2). The sequencing results were de-multiplexed and processed by CLC Genomics Workbench (CLC Bio). A total of 113,065,267 to 763,831,282 raw reads were generated for each replicate and were exported in the FASTQ format for the data upload. The reads were then mapped to the PhiX control sequences (NCBI Genbank accession number: NC_001422) to eliminate the PhiX control reads with the following parameters: mismatch cost: 2; insertion cost: 3; deletion cost: 3; length fraction: 0.9; similarity fraction: 0.9; and non-specific matches were randomly mapped. A total of 112,376,633 to 661,109,040 reads were unmapped. As these reads were sequenced from the 5′ end of the enriched RPF to 50 bp downstream, which is longer than the size-selected RPF (26 to 34 bp), the 5′ end sequences of the 3′ adapter sequence of the NEBNext Multiplex Small RNA Library Prep Set for Illumina (NEB) were also included. To remove the adapter sequences from the reads prior to mapping, the sequences were trimmed by the following parameters: action: remove adapter; strand: minus; mismatch cost: 2; gap cost: 3; internal match minimum score: 3; and end match minimum score: 3. Ultimately, the removed adapter sequence was 5′−ATACGAGATNNNNNNCGTGACTGGAGTTCAGACGTGTGCTCTTCCGATCTT−3′, in which the NNNNNN sequences were CACTGT, ATTGGC, TACAAG, and TTTCA for index 5, 6, 12, and 19, respectively. The reads were additionally trimmed based on their overall quality (score: 0.05, maximum ambiguous nucleotides: 2) and length (>15 bp). The trimming steps yielded 90.68 to 98.47% of the PhiX control unmapped reads. To confirm the data quality and reproducibility of the reads to analyze the translational abundance of the genes, the reads were mapped to their genome sequence. A total of 84,947,464 to 590,644,871 reads with an average read length of 25.8 to 33.7 bp were mapped with random mapping of non-specific matches, while 1,833,155 to 103,819,037 reads with an average read length of 25.8 to 33.1 bp were mapped with ignored mapping of non-specific matches (mismatch cost: 2; insertion cost: 3; deletion cost: 3; length fraction: 0.9; similarity cost: 0.9). The overall statistics of the data processing are summarized in Table 2. The mapped information was exported in a BAM file format, and the number of mapped reads at each genomic position was counted as the read count. Normalized read value and principal component analysis (PCA) plots were generated using the DESeq. 2 package in R^34^.

**Table 2:** Overall statistics of ribosome profiling data.

Species	Growth phase	Number of raw reads	Number of PhiX_unmapped read	Number of trimmed_read	Trimmed reads length (bp)	Number of randomly mapped reads	Number of uniquely mapped reads	Uniquely mapped read length (bp)	Number of mapped reads within CDS	Raw read FASTQ accession
*S. avermitilis* MA-4680	E1	269,943,816	213,953,123	209,920,058	29.3	203,095,358	12,355,916	29.9	7,361,468	SRP158023
	E2	219,153,779	155,106,616	150,989,307	32.2	115,492,305	2,022,429	32.7	1,638,497	
	T1	230,159,662	148,885,319	144,318,010	32.7	119,536,793	3,541,737	32.8	2,455,564	
	T2	266,436,355	175,849,102	170,463,269	31.5	139,053,054	2,304,886	32	1,665,882	
	L1	315,653,269	223,796,896	217,299,279	32.5	171,944,766	7,386,273	31.7	4,018,623	
	L2	308,070,582	228,756,230	221,698,272	31.5	169,555,584	2,929,594	31.8	2,099,075	
	S1	353,167,764	253,272,121	245,481,924	32.7	184,855,688	13,061,116	29.6	3,594,618	
	S2	314,771,387	223,201,435	216,515,335	32.3	169,100,733	7,789,273	29.6	2,170,481	
*S. clavuligerus* ATCC27064	E1	295,724,334	202,630,787	196,272,522	30.1	186,099,317	80,030,583	29.6	8,017,879	SRP188290
	E2	307,178,979	220,741,829	200,168,124	25.9	187,152,134	61,281,649	25.8	10,793,485	
	T1	253,508,213	169,638,278	162,668,883	29.5	153,361,820	89,299,628	29.2	6,590,232	
	T2	278,275,008	192,923,207	186,424,116	29.5	175,804,007	87,504,701	29.1	6,622,288	
	L1	270,412,414	177,901,515	172,769,472	29.5	157,803,554	87,274,405	29.3	5,179,486	
	L2	247,353,047	173,740,843	167,591,084	29.3	153,762,950	80,385,683	29.1	4,290,733	
	S1	238,332,934	174,931,608	168,131,662	29.2	151,884,988	85,485,768	29	6,272,720	
	S2	265,467,800	177,945,831	170,844,372	29.3	158,910,658	87,634,498	29.1	8,614,984	
*S. lividans* TK24	E1	309,069,871	221,703,188	211,211,182	29.6	199,318,423	97,211,458	29.6	19,459,202	PRJEB31507
	E2	296,200,898	195,130,032	185,499,278	30.7	173,681,372	81,149,257	30.4	13,680,737	
	T1	275,143,588	188,420,163	183,178,560	29.6	173,135,378	24,771,546	29.1	7,284,890	
	T2	212,032,571	140,458,753	136,973,078	31.8	125,039,755	21,109,391	30.4	7,941,585	
	L1	263,274,610	144,638,209	142,426,169	31.8	113,452,166	9,735,525	31.2	6,343,211	
	L2	224,511,134	154,437,906	150,790,276	31.9	137,882,471	19,449,401	31	13,544,509	
	S1	181,850,628	120,826,462	116,547,304	32.2	96,190,179	10,046,165	30.9	7,412,208	
	S2	297,413,784	249,272,969	244,109,074	32.7	233,266,663	13,457,825	33.1	8,447,573	
*S. venezuelae* ATCC15439	E1	631,858,582	536,439,531	522,569,147	33.2	489,456,639	69,000,627	31.5	5,079,524	SRX6932518 ~ SRX6932525
	E2	535,926,210	429,105,453	415,708,343	33.8	390,659,798	40,255,232	31.8	3,642,920	
	T1	394,870,178	340,483,910	329,759,627	32.4	300,485,612	40,691,934	30.9	2,723,458	
	T2	166,241,490	161,092,945	157,601,248	31.8	138,943,001	35,483,940	30.6	2,162,631	
	L1	763,831,282	661,109,040	641,879,092	32.2	590,644,871	71,846,636	30.7	5,611,836	
	L2	646,261,568	533,891,315	520,671,902	32	482,564,893	67,853,120	30.9	4,261,890	
	S1	451,939,879	378,474,029	369,204,078	31.6	297,503,315	52,715,125	30.6	3,509,283	
	S2	168,577,692	164,169,059	158,023,893	31.1	147,637,916	34,179,596	30.4	1,764,845	
*S. tsukubaensis* NRRL 18488	E1	125,024,824	123,919,014	121,549,761	30.1	102,297,821	2,307,786	30.7	1,572,115	SRP103795
	E2	124,528,713	123,522,173	120,322,929	30	97,672,956	1,833,155	31.4	1,313,616	
	T1	132,160,059	131,056,038	126,769,305	31.1	99,903,619	8,779,981	29.7	2,895,583	
	T2	113,065,267	112,376,633	109,608,582	30.6	84,947,464	2,814,409	29.5	1,142,882	
	L1	162,942,510	161,871,882	157,083,409	30.7	137,909,625	52,297,448	29.3	9,275,687	
	L2	166,664,595	165,825,698	161,666,872	30.9	140,291,702	56,575,985	29.1	7,533,975	
	S1	146,036,258	144,790,983	138,367,948	29.7	115,443,226	6,902,386	29.4	1,325,229	
	S2	199,958,654	199,442,987	192,788,031	30	171,209,443	103,819,037	28.9	4,429,568	

## Data Records

Raw read FASTQ files, trimmed read FASTQ files, mapped read BAM files, and the gene expression text files of all samples were uploaded to the public databases (Tables 1 and 2). Raw read FASTQ files of RNA-Seq and ribosome profiling of three species (*S. avermitilis*, *S. clavuligerus*, *S. tsukubaensis*) were deposited at the National Center for Biotechnology Information Sequence Read Archive (NCBI SRA)^35–37^. Raw read FASTQ files of RNA-Seq and ribosome profiling of *S. lividans* were deposited at the European Nucleotide Archive (ENA)^38^. Raw read FASTQ files of RNA-Seq of *S. venezuelae* were deposited at the ENA^39^. Raw read FASTQ files of ribosome profiling of *S. venezuelae* were deposited at the NCBI SRA^40–47^. Trimmed read FASTQ files and mapped read BAM files of the raw read FASTQ files in the NCBI SRA (RNA-Seq and ribosome profiling data of *S. avermitilis*, *S. clavuligerus*, *S. tsukubaensis*, and ribosome profiling data of *S. venezuelae*) were deposited at the ENA with a new accession^48^. Trimmed read FASTQ files and mapped read BAM files of the raw read FASTQ files in the ENA (RNA-Seq and ribosome profiling data of *S. lividans*, and RNA-Seq data of *S. venezuelae*) were also deposited at the ENA with the same accession as each corresponding raw read FASTQ file^38,39^. The gene expression profile as raw read counts of RNA-Seq and ribosome profiling data of *S. avermitilis*^49^, *S. clavuligerus*^50^, *S. tsukubaensis*^51^, and ribosome profiling data of *S. venezuelae*^52^ are available in a text file format in the Gene Expression Omnibus (GEO) database. Also, the gene expression profiles of all datasets (RNA-Seq and ribosome profiling of the five species), including raw read counts, DESeq. 2 normalized values, fold change values between growth phases, and *p*-values for the fold changes, are available in a text file format in the Figshare^53^. The raw read FASTQ data of *S. clavuligerus* in NCBI SRA^36^ was published in the previous study^21^. Also, the raw read FASTQ data of *S. lividans* in ENA^38^ was published in the previous study^22^. Note that the ribosome profiling data of *Streptomyces griseus* was uploaded under the same accession with those of *S. venezuelae*, but they are not described in this study.

## Technical Validation

### RNA-Seq read quality validation

**Fig. 2:**
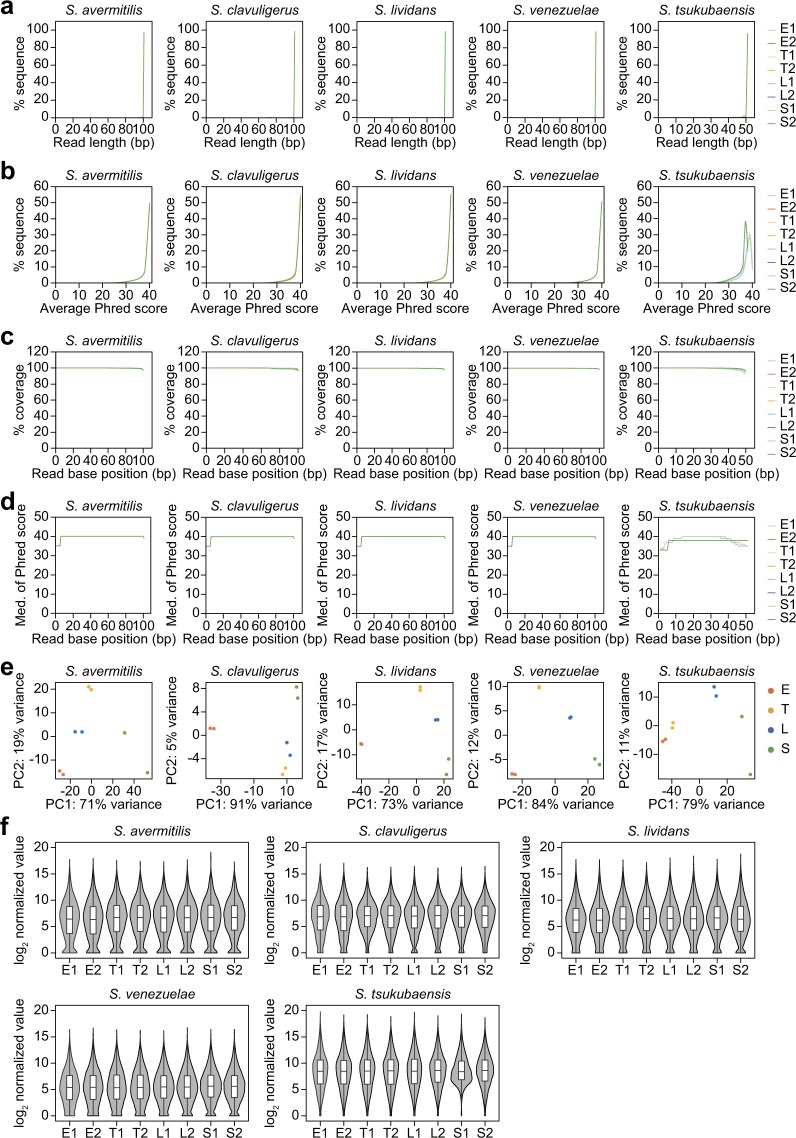
Read quality analysis of RNA-Seq samples of five *Streptomyces* species at four growth phases. The replicate of each growth phase is represented as “1” or “2” after the growth phase. (**a**) Read length distribution of trimmed reads. (**b**) Distribution of average Phred scores of the trimmed reads. (**c**) The number of sequences that cover individual base positions normalized to the total number of sequences at each base position. (**d**) The distribution of the median Phred quality scores that were observed at each base position. (**e**) PCA plot of RNA-Seq mapped reads of each gene. (**f**) Violin and box plot of the log_2_ normalized expression values.

A total of 40 RNA-Seq runs that were applied to five species at four time points as duplicates yielded on average 16,430,039 reads (*S. avermitilis*), 13,961,155 reads (*S. clavuligerus*), 15,686,025 reads (*S. lividans*), 12,899,493 reads (*S. venezuelae*), and 51,144,650 reads (*S. tsukubaensis*). After trimming the sequencing reads by quality score and nucleotide length, more than 99.8% of the sequencing reads remained, which indicated high-sequencing quality. The remaining reads were used as input to generate sequencing QC reports in the CLC Genomics Workbench to validate the quality of the reads. At first, the overall read lengths were extremely long, corresponding to the sequencing read recipe (Fig. 2a, Table 1). For the four species that were sequenced with the 100-bp read recipe, the percentage of read lengths that were over 100 bp was more than 97.9%, and for *S. tsukubaensis*, which was sequenced with the 50-bp read recipe, the percentage of read lengths that were over 50 bp was more than 93.8%. Further, more than 98.6% (*S. avermitilis*), 98.9% (*S. clavuligerus*), 98.9% (*S. lividans*), 98.9% (*S. venezuelae*), and 96.4% (*S. tsukubaensis*) of the total reads exhibited an average Phred score of greater than 30, which indicates 99.9% base call accuracy (Fig. 2b). In addition, the quality of each base of the obtained reads was examined. The overall base positions of the sequencing reads were highly covered, and even the lowest average values of the coverage were 97.5% (*S. avermitilis*), 97.6% (*S. clavuligerus*), 97.8% (*S. lividans*), 98.3% (*S. venezuelae*), and 95.4% (*S. tsukubaensis*) at the last position, respectively (Fig. 2c). Moreover, the median values of the Phred scores per base position of the reads were consistently high across reads, with 40 scores in four species and 38 scores in *S. tsukubaensis* (Fig. 2d). From these quality validation results, we validated the quality of all obtained RNA sequencing reads for subsequent analysis.

### Assessment of transcriptome data

The qualified reads were mapped to each reference genome with a uniquely mapped percentage that ranges from 76.91% to 95.09% (*S. avermitilis*), 52.19% to 90.88% (*S. clavuligerus*), 60.15% to 87.08% (*S. lividans*), 67.35% to 86.23% (*S. venezuelae*), and 76.33% to 96.26% (*S. tsukubaensis*) **(**Table 1**)**. The number of uniquely mapped reads at each gene was counted and normalized using the DESeq. 2 package in R^33^ to reduce variation between samples. Using the normalized values, principal component analysis (PCA) was performed, which validated the high reproducibility of the sequencing data (Fig. 2e). The distribution of log_2_ (DESeq normalized value + 1) broadly ranged from 0 to 20 in the different growth phase samples (Fig. 2f).

### Ribosome profiling read quality validation

**Fig. 3:**
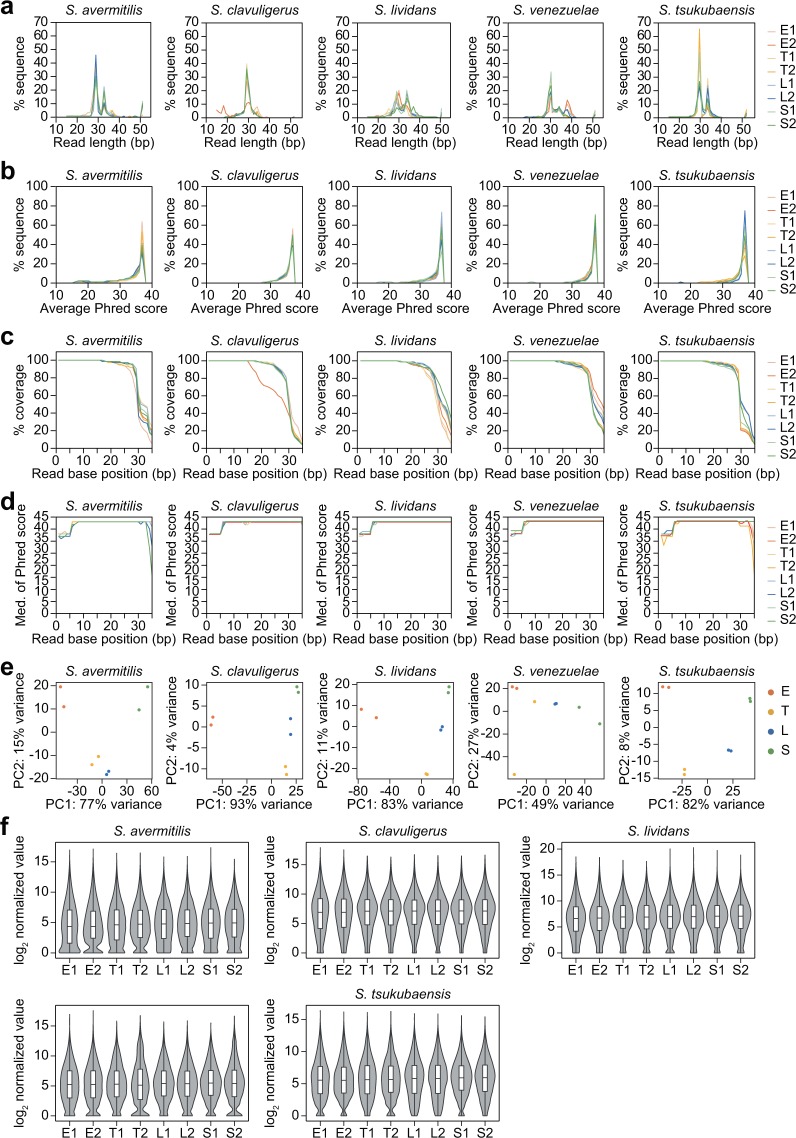
Read quality analysis of ribosome profiling samples of five *Streptomyces* species at the four growth phases. The replicate of each growth phase is represented as “1” or “2” after the growth phase. (**a**) Read length distribution of trimmed reads. (**b**) Distribution of average Phred scores of the trimmed reads. (**c**) The number of sequences that cover individual base positions normalized to the total number of sequences at each base position. (**d**) The distribution of median Phred quality scores that were observed at each base position. (**e**) PCA plot of ribosome profiling mapped reads of each gene. (**f**) Violin and box plot of the log_2_ normalized expression values.

A total of 40 ribosome profiling reads were obtained from five *Streptomyces* species at four time points as duplicates. Unlike the RNA-Seq data, the trimmed reads were considered as raw sequences of the enriched RPF sequences, as additional PhiX control and adapter sequences that were involved in the ribosome profiling steps must be removed (Fig. 1a,b). Since the RPF fragments were selected by size, ranging from 26 to 34 bp, the 3′ end of the total 50 bp sequencing read contained non-RPF sequences, such as the 3′ adapter sequences, which do not represent the quality of the RPF reads. Thus, the QC reports on the trimmed reads were exported from CLC Genomics Workbench (Qiagen) to assess the quality of the RPF reads. The read length distribution exhibited a broad range from 20 to 40 bp, with one or two enriched peaks (Fig. 3a). The enriched peak sizes were comparable to the monosome-protected sizes, and they varied for different species, while they were more conserved for different growth phase samples of the same species. The differences in RNA degradation efficiency of RNase I or MNase across species may be the primary reason for the observed size differences^54^. Further, the read quality that was measured by the average Phred scores was generally high in all samples of the five species; the quality of more than 94% of the reads was higher than Q20, and more than 80% were higher than Q30 (Fig. 3b). Both per-sequence and per-base analyses of the read quality were observed. As the read size ranged mostly between 25 and 35 bp after adapter trimming, the base number coverage at each position of the 50 bp read dramatically decreased at the 3′ end (Fig. 3c). In terms of species, most of them exhibited the highest decline at 28 to 30 bp, which was consistent with the read length distribution (Fig. 3a). Given the base coverage, the median Phred score per base was demonstrated to be from 1 to 35 bp (Fig. 3d). The overall median of the quality score was approximately Q38, while the median score at the 5′ end was slightly lower than that of the middle section, and the score at the 3′ end of select species showed dramatic reductions. The low quality at the 3′ end may be due to some portions of identical long reads, which were somehow enriched during the size selection step of library construction, which stimulates wrong base calling. For the *S. clavuligerus* E2 sample, enriched peaks of less than 20 bp in length for approximately 10% of the total reads were unexpectedly found, along with decreased coverage at 15 bp, but these peaks did not seem to affect the overall read quality (Figs. 3a,c,d). Overall, most of the reads were shorter than 35 bp, and the read quality of all samples was high and suitable for downstream analyses.

### Assessment of translatome data

To examine the additional quality of the reads for the translational abundance of each gene, the trimmed reads were mapped to their corresponding genome. Based on the mapping parameter, some reads would be non-specifically aligned to more than one genomic position due to highly repetitive genomic regions, including rRNA genes. Approximately, 43.1 to 590.6 million reads (75.3 to 96.8% of the trimmed reads) were mapped when the non-specifically matched reads were randomly assigned to one of the mapped positions, while 1.8 to 103.9 million reads (1.3 to 54.9% of the reads) were uniquely mapped when the non-specifically matched reads were excluded (Table 2). These results suggest that the non-specifically matched reads were generally more than half of the total mapped reads. Further, the proportion of these reads varied in different samples even within the same species, which is because the rRNA was enriched during the monosome recovery step, and the efficiency of rRNA removal differed across samples^55^. *S. clavuligerus* showed the highest uniquely mapped read number and ratio among five species, with an average of 82.4 million reads (47% of the trimmed reads). The *S. clavuligerus* E2 sample showed a relatively lower mapped number (61.3 million reads, 30.6% of the trimmed reads) compared to other *S. clavuligerus* samples. *S. venezuelae* showed 34.2 to 69 million mapped reads (average 13.9% of the trimmed reads), respectively. The two early samples of *S. lividans* showed 97.2 and 81.1 million mapped reads (46 and 43.8% of the trimmed reads), respectively, while other samples showed low numbers (9.7 to 24.8 million mapped reads, 5.5 to 15.4% of the trimmed reads). *S. tsukubaensis* showed various ranges for the mapping read number; L2 and S2 samples showed 56 and 103 million mapped reads (35 and 53.9% of the trimmed reads), respectively, while other samples showed a lower number of mapped reads (1.9 to 8.8 million mapped reads, 1.5 to 6.9% of the trimmed reads). *S. avermitilis* showed the lowest number of mapped reads and ratio among the five species, with 2 to 13.1 million mapped reads (1.3 to 5.9% of the trimmed reads). Although the minimum mapped read number among the samples was 1.8 million, the numbers obtained are, based on several bacterial transcriptome studies, considered sufficient for analysis of the whole translational profile and differential expression levels of genes, as 1 to 5 million reads are suggested for high statistical significance^56–59^. Among the uniquely mapped reads, some reads were mapped within RNA genes, rather than protein-coding genes, which mostly corresponded to tRNA genes. These reads may be the fragments of tRNA and rRNA that were bound to the ribosome and then enriched during monosome recovery^60^. Therefore, further validation was performed using only the mapped reads of the protein-coding genes. A total of 1.1 to 19.5 million reads were mapped to protein-coding genes that were 4.3 to 81.0% of the uniquely mapped genes, which indicates a high ratio of tRNA gene-mapped reads (Table 2). To validate the mapped read quality, the reproducibility of the mapped read number among biological replicates was investigated by PCA. All replicates were found to exhibit high reproducibility (Fig. 3e). The mapped read quality for quantitative analysis, such as the differential translational abundance of genes during growth, was examined by the distribution of the normalized values at four different growth phases, as described in the “Methods” section. The overall log_2_ value (DESeq normalized value + 1) broadly ranged from 0 to 20, which was considered significant to analyze the translational abundance in different growth phases (Fig. 3f). In conclusion, the mapped reads were confirmed to exhibit high quality in terms of sequencing depth, reproducibility, and translational abundance.

## Data Availability

Versions and parameters of all the bioinformatic tools that were used in this work are described in the “Methods” section.
