# Author Correction: Transcriptome and translatome profiles of Streptomyces species in different growth phases

**DOI:** 10.1038/s41597-021-00858-2

**Published:** 2021-02-26

**Authors:** Woori Kim, Soonkyu Hwang, Namil Lee, Yongjae Lee, Suhyung Cho, Bernhard Palsson, Byung-Kwan Cho

**Affiliations:** 1 Department of Biological Sciences and Ki for the Biocentury, Korea Advanced Institute of Science and technology, Daejeon, 34141 Republic of Korea; 2 Department of Bioengineering, University of California San Diego, La Jolla, CA 92093 USA; 3 Department of Pediatrics, University of California San Diego, La Jolla, CA 92093 USA; 4 Novo Nordisk Foundation Center for Biosustainability, Technical University of Denmark, Lyngby, 2800 Denmark; 5 Intelligent Synthetic Biology Center, Daejeon, 34141 Republic of Korea

**Keywords:** Prokaryote, Next-generation sequencing, RNA sequencing

Correction to: *Scientific Data* 10.1038/s41597-020-0476-9, published online 08 May 2020

Following publication, it was found that two accession numbers given in the manuscript text were incorrect. A figure label was also missing from Figure 3f.

Both the HTML and PDF versions have been updated to reflect these changes.

